# Downregulation of miR-29c-3p is associated with a poor prognosis in patients with laryngeal squamous cell carcinoma

**DOI:** 10.1186/s13000-019-0893-2

**Published:** 2019-10-15

**Authors:** Ruihua Fang, Yongjin Huang, Jinghua Xie, Jianzhong Zhang, Xiaobin Ji

**Affiliations:** 1 Department of Otolaryngology, Guangzhou First People’s Hospital, Guangzhou Medical University, Guangzhou, 510180 Guangdong People’s Republic of China; 2 Department of Otolaryngology, The Fifth Affiliated Hospital, Guangzhou Medical University, Guangzhou, Guangdong 510700 People’s Republic of China

**Keywords:** Laryngeal squamous cell carcinoma, Prognosis, Biomarker, MicroRNA, MiR-29c-3p

## Abstract

**Background:**

Laryngeal squamous cell carcinoma (LSCC) is considered to be a common malignancy of the head and neck with poor prognosis for its late diagnosis, metastasis and recurrence. Growing evidence demonstrates that the dysregulation of miR-29c-3p (microRNA-29c-3p) plays an important role in various tumor processes. Our study investigates the expression of miR-29c-3p in LSCC and analyzes the correlation of its dysregulation with clinicopathologic parameters and prognosis.

**Methods:**

The expression of hsa-miR-29c-3p in LSCC tissues and the adjacent normal laryngeal tissues was detected in 96 LSCC formalin-fixed paraffin-embedded tissues by quantitative real-time PCR (qRT-PCR). The SPSS statistical software package (17.0) was used to analyze the associations between miR-29c-3p expressions and various clinicopathological characteristics. The overall survival (OS) was analyzed by the Kaplan-Meier method and log-rank test, and we analyzed the independent factor of prognosis by Cox proportional hazard analysis.

**Results:**

A downregulation of miR-29c-3p expression in LSCC was significantly correlated with smoking index, tumor size, tumor site, differentiation, T classification, TNM stage, and lymph node metastasis (*P* < 0.05), but there was no correlation with age and alcohol consumption (*P* > 0.05). In the multivariate survival analysis, low miR-29c-3p expression was associated with shorter overall survival (*P* < 0.05). Furthermore, miR-29c expression was an independent prognostic factor for laryngeal cancer patients.

**Conclusions:**

MiR-29c-3p has different expression levels at different stages of tumor progression, suggesting that miR-29c-3p may be a promising biomarker for evaluating the progression of LSCC and the prognosis of patients with LSCC. MiR-29c-3p can also be a novel molecular target for anti-laryngeal cancer therapy.

## Introduction

Laryngeal squamous cell carcinoma (LSCC), as the most common histopathological type of laryngeal cancer, is the second-most common malignancy of the head and neck [1, 2].

According to studies, almost 3650 people died from LSCC worldwide in 2012, concomitantly with the increasing incidence and mortality of LSCC year-by-year [3]. At present, surgery, radiotherapy and chemotherapy are the main treatments for LSCC, but these treatments cannot meet the requirements for reducing the morbidity and mortality of LSCC. Moreover, the 5-year survival rate of advanced patients was less than 50%, mainly due to metastasis and recurrence [4]. Most patients already have advanced stage LSCC because of the lack of Early diagnosis. In addition, accumulating studies have proved miRs can serve as potential diagnostic biomarkers for laryngeal cancer [5]. Therefore, exploring novel biomarkers for the early stage diagnosis, prognosis and treatment of LSCC patients has been in high demand.

MiRNAs, 19 to 24 nucleotides in length, are endogenous noncoding single-stranded small RNA molecules that exist in many organisms and regulate gene expression at the posttranscriptional level by binding the 3′-untranslated region (3′-UTR) of target mRNAs through complete or incomplete complementary seed sequences and assembling in RNA-induced silencing complex (RISC) [6–8]. Evidently, miRNAs play important roles in all tumor-related processes, including proliferation, differentiation, apoptosis, metastasis, invasion and angiogenesis in LSCC.

MiR-29c-3p, as a tumor suppressor in the miRNAs family [9], has been demonstrated to be downregulated in many solid tumors, such as nasopharyngeal carcinoma [10], gastric cancer [11], hepatocellular carcinoma [12], gallbladder cancer [13], esophageal cancer [14], breast cancer [15], colon cancer [16] and head and neck cancers [17], which also revealed that lower expression of miR-29c was associated with lymph node metastasis, tumor differentiation, TNM stage and poor prognosis. Study has revealed that miR-29s  group were frequently downregulated in head and neck squamous cell carcinoma (HNSCC), such as tongue squamous cell carcinoma and hypopharyngeal squamous cell carcinoma, which can normally inhibit cancer cell migration and invasion through their regulated pathways [18]. Besides, numerous studies have showed that miR-29c can inhibit the proliferation, invasion and metastasis of tumors and promote apoptosis by regulating a variety of oncogenes, cell pathways, cell cycle and epithelial to mesenchymal transition (EMT) [7, 19–21]. In contrast, decreased expression of miR-29c was associated with a poor prognosis [22] and can even be used as a novel marker for cancer metastasis [17], drug treatment [7] and prognosis [13]. A recent study has demonstrated that miRNA arrays may contribute to the early diagnosis of LSCC, as well as the detection of clinical stage and prognosis [5]. Ayaz et al. found miR-331-3p, 603, 1303, 660-5p and 212-3p were not expressed in healthy individuals and patients with any other diseases but in LSCC, which suggessted that they could serve as novel non-invasive biomarkers for diagnosis of LSCC [23]. However, the role of miR-29c in LSCC is still unclear and lacks any relevant studies.

In the present study, we determined and compared the expression of miR-29c in LSCC tissues and adjacent normal laryngeal tissues. Furthermore, we evaluated the relationships between miR-29c expression and the clinical parameters and the survival of LSCC patients and revealed the prognostic factors of LSCC via multivariate Cox hazard regression analysis.

## Methods

### Patients and clinical specimens

Sixty-six cases of LSCC tissue specimens and 30 cases of adjacent normal laryngeal tissue specimens were obtained from the paraffin-embedded tissues of patients who underwent laryngeal surgery at the Department of Otorhinolaryngology, Guangzhou First People’s Hospital, Guangzhou Medical University, between the year 2008 and 2012. All patients were pathologically diagnosed with LSCC by two senior pathologist, excluding those that received chemotherapy, radiotherapy, hormone therapy or immunotherapy before surgery.

Of the 66 LSCC patients, 62 were male and 4 were female, with a mean age of 60.6 ± 6.85 years. All of the patients were reviewed and the following clinicopathologic parameters were collected: age at diagnosis, sex, smoking index (number of cigarettes per day·years), alcohol consumption, size and site of tumor, differentiation, T classification, TNM stage, OS, and lymph node metastasis, as shown in Table 1.

**Table 1:** Relationship between miR-29c-3p and clinicopathological parameters in 66 patients with LSCC

Clinicopathologic parameters	Value, No.(%)	miR-29c-3p expression ($\overline{X}\pm S$)	*P*-value
Gender			
Male	62 (93.94)	2.61 ± 0.28	0.855
Female	4 (6.06)	2.59 ± 0.30	
Age			
≤ 60 y	26 (39.39)	2.62 ± 0.30	0.904
> 60 y	40 (60.61)	2.61 ± 0.27	
Drinking			
Yes	21 (31.82)	2.53 ± 0.30	0.118
No	45 (68.18)	2.64 ± 0.27	
Smoking index			
< 400	30 (45.45)	2.72 ± 0.25	0.002
≥ 400	36 (54.55)	2.51 ± 0.27	
Tumor size			
≤ 3 cm	38 (57.58)	2.68 ± 0.25	0.011
> 3 cm	28 (42.42)	2.50 ± 0.29	
Tumor site			
Supraglottic	19 (28.79)	2.47 ± 0.23	0.010
Glottic	45 (68.18)	2.68 ± 0.28	
Subglottic	2 (3.03)	2.39 ± 0.06	
Differentiation			
Well	36 (54.55)	2.69 ± 0.28	0.029
Moderate	23 (34.85)	2.54 ± 0.25	
Poor	7 (10.60)	2.43 ± 0.24	
Lymph node metastasis			
N0	36 (54.55)	2.71 ± 0.28	0.001
N+	30 (45.45)	2.49 ± 0.24	
T classification			
T1	7 (10.61)	2.89 ± .0.18	0.001
T2	14 (21.21)	2.74 ± .0.22	
T3	13 (19.70)	2.57 ± .0.65	
T4	32 (48.48)	2.51 ± 0.27	
TNM stage			
I	7 (10.61)	2.89 ± 0.18	0.004
II	13 (19.70)	2.72 ± 0.25	
III	14 (21.21)	2.57 ± 0.24	
IV	32 (48.48)	2.52 ± 0.28	

One-way ANOVA was used to analyze the correlation between the expression of miR-29c and clinicopathological parameters of the patients.

*indicates *P* < 0.05.

It was confirmed in a pathological report that no cancer cells were found in the 30 adjacent normal laryngeal tissues, which were more than 2 cm from the edge of the tumor.

### Quantitative real-time polymerase chain reaction (qRT-PCR)

MiRNA was extracted from formalin-fixed, paraffin-embedded (FFPE) tissues using an FFPE miRNA Purification Kit (Tiangen, Beijing, China). Reverse transcription was performed using a miRcute enhanced miRNA cDNA synthesis kit (Tiangen, Beijing, China). The expression level of miR-29c-3p was quantified by qRT-PCR using miRcute Enhanced miRNA Fluorescence Quantification Kit (SYBR Green) (Tiangen, Beijing, China), which was normalized to U6. The relative miR-29c expression was analyzed using the formula 2^-△Ct^, △Ct = Ct (miR-29c) – Ct (U6).

### Statistical analysis

SPSS19.0 software (SPSS Inc., IL, USA) and GraphPad Prim 7 were used for statistical analysis. All data was converted into log10 + 5 variables, and the converted data conformed to the normal distribution, which were expressed as the mean ± standard deviation (SD). The t-test was applied to test the differential expression of miR-29c in LSCC tissues compared to adjacent nonmalignant tissues. One-way ANOVA was used to determine the relationships between miR-29c expression and clinicopathological characteristics. The overall survival time was calculated on a monthly basis, and the survival analysis was done by the log- rank test. Risk factors of LSCC were analyzed by univariate and multivariate analyses on the basis of Cox proportional hazards model. *p* < 0.05 was considered statistically significant, and *p* < 0.01 was considered very significant.

## Results

### Downregulation of miR-29c in LSCC

**Fig. 1:**
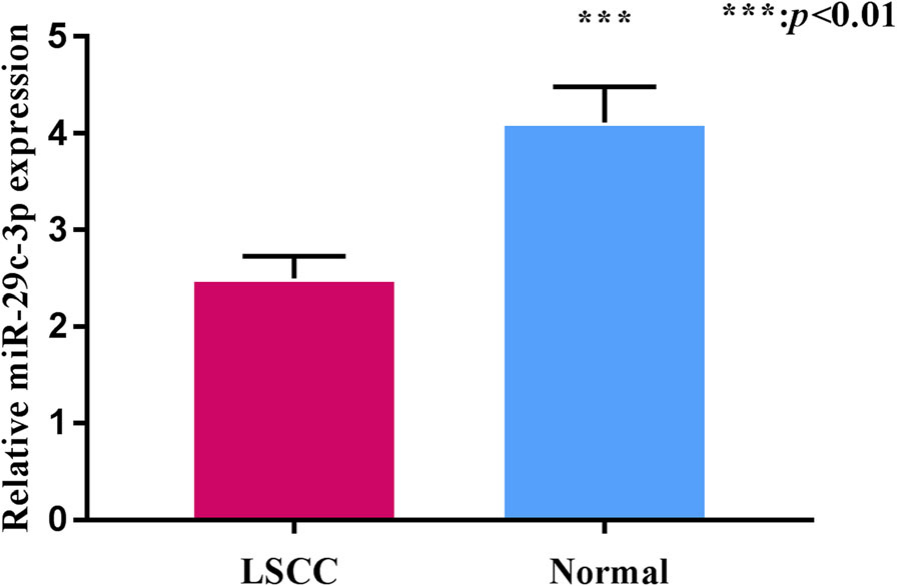
Relationship between miR-29c-3p expression and clinicopathologic parameters in LSCC

The results of qRT-PCR showed that the relative expression level of miR-29c in the 66 patients with LSCC was 2.60 ± 0.29, which was significantly lower than that in the 30 adjacent normal tissues (4.11 ± 0.36) (*P* < 0.01). This result indicated that miR-29c was downregulated in LSCC, suggesting that miR-29c might play an anti-oncogenic role in LSCC (Fig. 1).

**Fig. 2:**
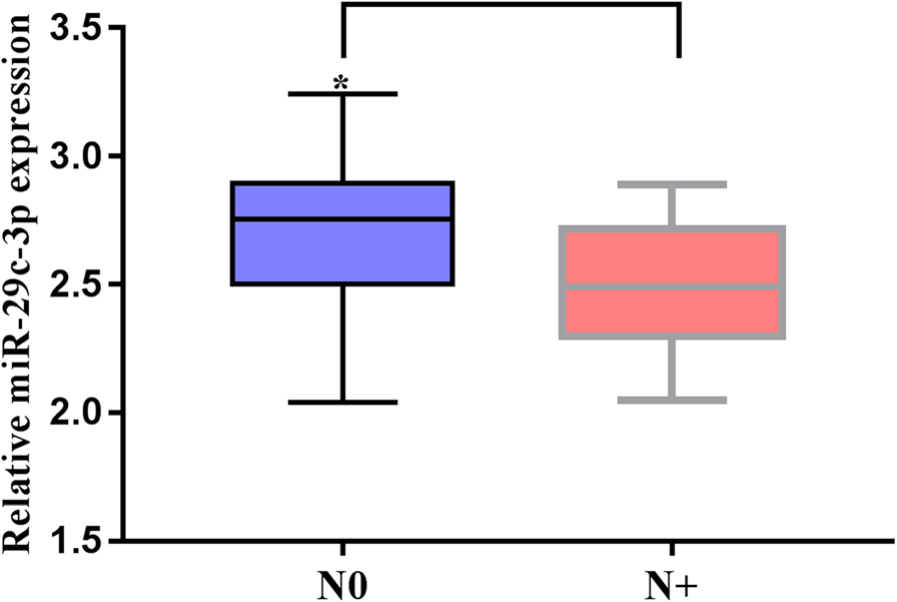
Relationship between miR-29c-3p and lymph node metastasis. *P < 0.05

As seen in Table 1, decreased miR-29c expression in LSCC tissue was significantly associated with smoking index, tumor size, tumor site, lymph nodes metastasis, tumor differentiation, T classification and TNM stage of LSCC (*P <* 0.05). In contrast, there was no significant correlations between miR-29c expression and age, gender or alcohol consumption (*P >* 0.05). The expression of miR-29c in patients with a smoking index < 400 and tumor diameter < 3 cm was significantly higher than that in patients with a smoking index ≥400 and tumor diameter ≥ 3 cm (*P* < 0.01). The expression level of miR-29c varied in patients with different tumor sites, with the highest expression of miR-29c in patients with glottic tumors, followed by glottic-type tumors, and the lowest in subglottic tumors (*P* < 0.05). It was also shown that patients without cervical lymph node metastasis had higher expression levels of miR-29c than patients with cervical lymph node metastasis (Fig. 2, *P* < 0.01). Furthermore, miR-29c overexpression was correlated with a lower degree of tumor differentiation and an advanced clinical stage (*P* < 0.01), suggesting that miR-29c might participate in the progression of the tumor.

### The expression levels of miR-29c are associated with patient prognosis in LSCC

**Fig. 3:**
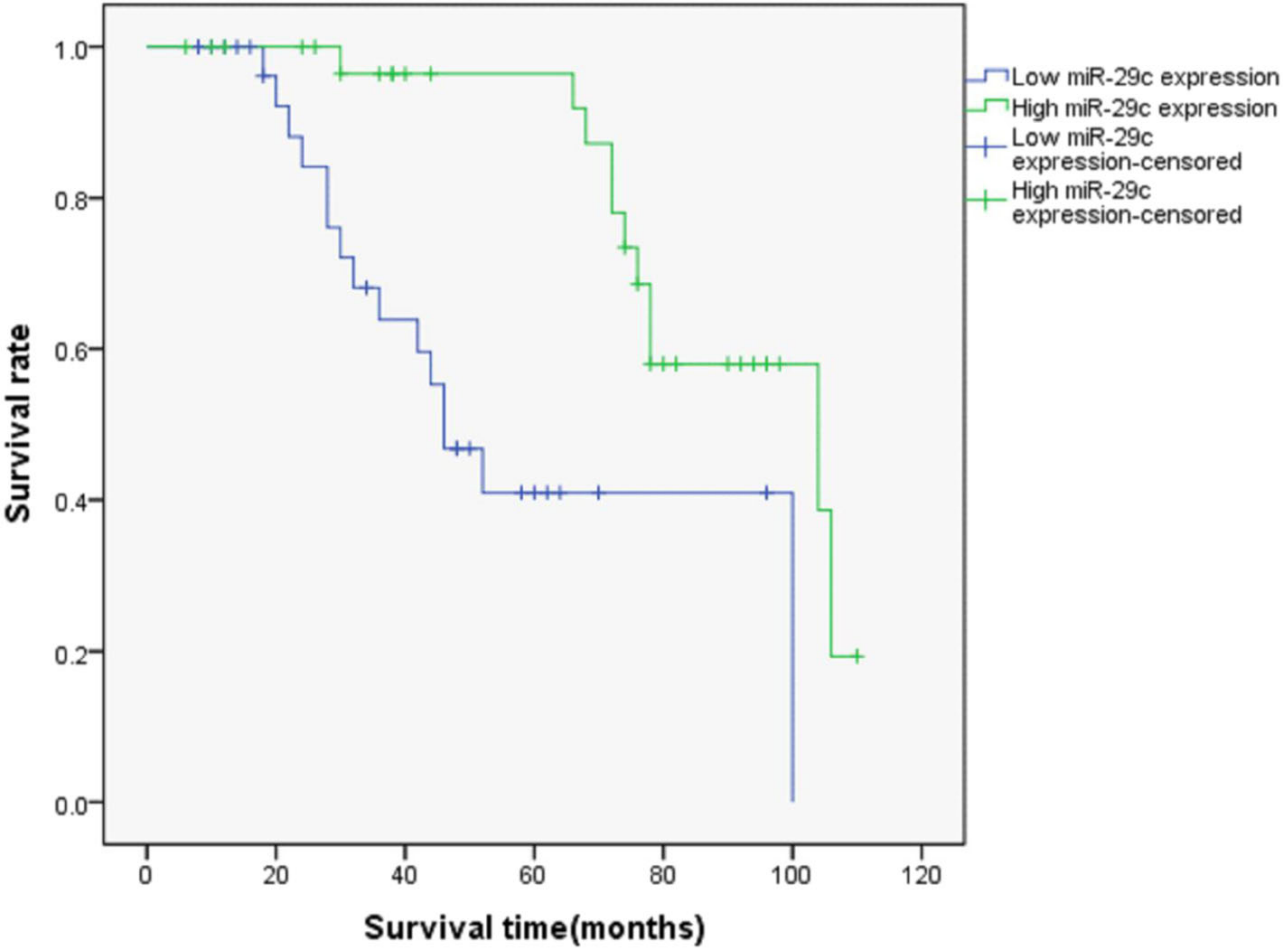
MiR-29c expression was relative to patient prognosis in LSCC. The survival rate in different miR-29c expression groups showed significant differences, *p* < 0.05

The average expression of miR-29c in the 66 cases of LSCC was used as the cut-point— ≥ 2.61 for the high expression group and < 2.61 as the low expression group. The Kaplan-Meier analysis showed that the high miR-29c expression group had a longer survival time than the low expression group, suggesting that miR-29c had the significance of prognostic evaluation (*p* < 0.01, Fig. 3). This result indicated that patients with a low expression level of miR-29c had a poor prognosis.

### MiR-29c was an independent prognostic factor for LSCC

The results of Univariate Cox hazard regression analysis showed that smoking index, tumor size, tumor site, lymph nodes metastasis, tumor differentiation, T classification and TNM stage and miR-29c expression were the factors impacting patient prognosis. In addition, the differentiation, lymph node metastasis, TNM stage and miR-29c expression were significantly correlated with patient survival by multivariate analysis (Table 2). Our study also demonstrated that patients with a high miR-29c level had a lower death risk, implying that the miR-29c expression level was an independent prognostic factor for laryngeal cancer, in addition to differentiation, lymph node metastasis and TNM stage.

**Table 2:** Univariate and multivariate Cox hazard regression analysis for patient prognosis in LSCC

Clinicopathologic parameters	Univariate analysis		Multivariate analysis	
	Hazard ratio (95% confidence interval)	*P*-value	Hazard ratio (95% confidence interval)	*P*-value
Gender	1.067 (0.139–8.00)	0.950		
Age	1.213 (0.521–2.824)	0.654		
Drinking	1.310 (0.579–2.960)	0.517		
Smoking index	0.397 (0.177–0.893)	0.026		
Tumor size	0.370 (0.166–0.84)	0.015		
Tumor site	2.495 (1.263–4.926)	0.008		
T classification	2.422 (1.486–3.947)	0.000		
Differentiation	2.620 (1.393–4.99)	0.003	3.578 (1.763–7.260)	0.000
Lymph node metastasis	7.157 (2.460–20.826)	0.000	3.931 (1.11–13.786)	0.033
TNM stage	2.462 (1.473–4.114)	0.001	2.262 (1.015–5.038)	0.046
miR-29c expression	0.215 (0.88–0.523)	0.001	.350 (0.129–0.949)	0.039

## Discussion

To date, the pathogenesis of laryngeal cancer is still unclear and the resistance to chemotherapy or radiotherapy account for frequent recurrent rate and high mortality. Therefore, it is vital to explore the molecular mechanisms and novel drug therapeutic targets of LSCC. In recent years, miRNAs have become a research hotspot because they are promising for use as biomarkers and drug therapeutic targets for various cancer diseases [24–26].

It was confirmed that among the expression profiles of 738 miRNAs in LSCC patients and healthy controls, 17 were upregulated and 9 were downregulated [5], but did not provide information about miR-29c. Our study first provided evidence that miR-29c was significantly downregulated in LSCC and played a critical role in cancer occurrence, which is consistent with the study on HNSCC [17].

In addition, smoking and drinking are considered to be risk factors in upper respiratory tract cancer (UADTC). It was reported that cigarettes can induce a large number of DNA mutations, leading to more DNA damage and making the risk of laryngeal cancer higher [27–29]. Our results also indicated that low level expression of miR-29c correlates with smoking index≥400, which implied that miR-29c might be involved in the pathomechanism of cigarettes inducing laryngeal cancer. Studies have also proven that overexpression of miR-29c inhibited the growth and metastasis of tumor cells and arrested tumor cells in the G1 phase, suggesting that miR-29c might be closely related to proliferation, cell cycle and apoptosis [30]. Our results demonstrated that the expression of miR-29c in tumors < 3 cm was higher than that in tumors ≥3 cm, which meant that miR-29c might regulate the proliferation and apoptosis of laryngeal carcinoma cells and further affect tumor size.

Furthermore, our results also indicated that the expression level of miR-29c in glottic laryngeal carcinoma was significantly higher than that in supraglottic and subglottic laryngeal carcinoma, and the subglottic laryngeal carcinoma had the lowest miR-29c expression. This may be associated with the fact that most glottic laryngeal cancer patients were diagnosed in the early stage of laryngeal cancer progression, when the expression level of miR-29c was also higher than that in advanced progression. Therefore, miR-29c might play a significant role in regulating the progression of laryngeal cancer.

Previous studies identified that miR-29c inhibited tumor invasion and metastasis by regulating different target genes and signaling pathways, such as MMP2 [31] and the MAPK pathway [13]. It has been emphasized that parecoxib is a potential drug for the treatment of digestive tract tumors, after a study found that parecoxib inhibited glioblastoma cell proliferation, migration and invasion by upregulating miR-29c [21]. In our study, we had demonstrated that miR-29c expression in laryngeal carcinoma patients without lymph node metastasis was significantly higher than that in patients with lymph node metastasis. Furthermore, a low level of miR-29c correlated with LSCC clinical stages, indicating that miR-29c might participate in laryngeal cancer cell migration and invasion, and increased miR-29c expression might be a novel treatment for laryngeal carcinoma.

Notably, Nygren et al [32] showed that a downregulation of miR-29c was associated with poor prognosis in breast cancer patients. Our present study demonstrated that low expression level of miR-29c in laryngeal cancer tissues significantly correlated with shorter survival time. By the multivariate Cox hazard regression analysis, we found that the miR-29c expression level was an independent prognostic positive factor for LSCC. A similar result was also found in pancreatic cancer patients [31].

In the future, we will pay more attention to the regulated mechanism of miR-29c in laryngeal cancer cell migration and invasion and explore a novel drug treatment for laryngeal cancer patients by increasing miR-29c expression.

## Conclusions

To our knowledge, the present study first reports that miR-29c-3p is downregulated in LSCC and low expression of miR-29c-3p is an independent predictor of poor prognosis for patients with LSCC. As a tumor suppressor, miR-29c may be a useful biomarker for tumor progression, early diagnosis and prognosis assessment. In the future, miR-29c may be a therapeutic drug target for laryngeal cancer.

## Data Availability

The data supporting the conclusions are included in the article. Raw data are available upon request.

## Abbreviations

3′-UTR: 3′- Untranslated region
CA19–9: Carbohydrate antigen19–9
CEA: Carcinoembryonic antigen
EMT: Epithelial to mesenchymal transition
FFPE: Formalin-fixed, paraffin-embedded
HNSCC: Head and neck squamous cell carcinoma
LSCC: Laryngeal squamous cell carcinoma
MiR-29c-3p: MicroRNA-29c-3p
OS: Overall survival
qRT-PCR: Quantitative real-time polymerase chain reaction
RISC: RNA-induced silencing complex
SD: Standard deviation
UADTC: Upper respiratory tract cancer
